# Association between thyroid function and obesity phenotypes in healthy euthyroid individuals: an investigation based on Tehran Thyroid Study

**DOI:** 10.1186/s40001-023-01135-1

**Published:** 2023-05-30

**Authors:** Behnaz Abiri, Amirhossein Ramezani Ahmadi, Maryam Mahdavi, Atieh Amouzegar, Majid Valizadeh

**Affiliations:** 1 Obesity Research Center, Research Institute for Endocrine Sciences, Shahid Beheshti University of Medical Sciences, P.O. Box 19395-476, Tehran, Iran; 2 Isfahan Endocrine and Metabolism Research Center, Isfahan University of Medical Sciences, Isfahan, Iran; 3 Endocrine Research Center, Research Institute for Endocrine Sciences, Shahid Beheshti University of Medical Sciences, Tehran, Iran

**Keywords:** Thyroid function, Free thyroxin, Metabolic phenotype, Obesity phenotype

## Abstract

**Aims:**

We investigated whether thyroid function could be associated with obesity phenotypes amongst euthyroid individuals.

**Materials and methods:**

A cross-sectional analysis was conducted among healthy, euthyroid subjects. The study participants were chosen from the Tehran Thyroid Study (TTS). We analyzed 2988 euthyroid adults and classified them into four obesity phenotype groups: metabolically healthy normal weight (MHNW), metabolically healthy obese (MHO), metabolically unhealthy normal weight (MUNW), and metabolically unhealthy obese (MUO). The statistical differences between thyroid hormones between various obesity phenotypes according to age and sex was compared using analysis of covariance (ANCOVA).

**Results:**

It was found that MHNW participants had higher levels of FT4 when compared with metabolically healthy or unhealthy obese subjects (*P* < 0.001), even after adjustment for the confounding variables. No difference was observed in the levels of TSH (*P* = 0.260) among obesity phenotypes. In the subgroup analysis according to the age, a significant difference was observed in the level of FT4 only in subjects with age < 55 years (*P* = 0.001). However, analyzing men and women separately did not show a significant difference in the FT4 level among obesity phenotypes (*P* > 0.05).

**Conclusion:**

“Metabolically abnormality” was independently related to low normal FT4 levels in overweight/obese euthyroid individuals. There is a need for further research to understand how low FT4 levels are linked to metabolically unhealthy states in euthyroid individuals.

## Introduction

The epidemic of obesity has become a major public health issue worldwide [1]. There are many complications associated with obesity, such as insulin resistance, dyslipidemia, hypertension, and systemic inflammation. These complications may eventually lead to type 2 diabetes, cardiovascular disease, and cancer [2–4].

The adverse effects of obesity on metabolic health are well known, but individual responses vary [5]. Metabolically healthy obese (MHO) individuals are those who are obese and have metabolic health [6]. In addition, metabolically unhealthy normal weight (MUNW) refers to a condition in which a person has abnormal metabolic parameters without being obese [7]. When combined with different metabolic profiles, obesity phenotypes are more accurate at predicting cardiovascular disease and mortality [8].

Thyroid hormones play a pivotal role in all major metabolic pathways, including protein, carbohydrate, and lipid metabolism, energy expenditure, and thermogenesis [1]. There is a strong link between thyroid hormones with obesity and metabolic disorders [9]. Research has shown an association between thyroid function, within a normal range, and anthropometric measurements and metabolic syndrome [10, 11]. This relationship has, however, been studied in limited studies with inconsistent results respect to different obesity phenotypes [12–14]. In light of this, it cannot be concluded with certainty whether minor changes in serum thyroid hormone levels are linked to obesity or metabolic diseases [1].

In general, these discrepancies suggest that the evidence is unclear about the relationship between thyroid hormone concentrations within the normal range and different metabolic phenotypes of obesity. Thus, this study was designed to clarify the relationship between thyroid hormone levels within the normal range and metabolic state in the general population, using a large sample in a cohort of the Tehran Thyroid Study (TTS).

## Methods and materials

### Study population

The study participants of this cross-sectional investigation were enrolled from the TTS [15], a cohort study that is conducted within the framework of the Tehran Lipid and Glucose Study (TLGS). The TLGS is a long-term, ongoing community-based research to identify and prevent noncommunicable disorders being carried out in district No. 13, located in the eastern part of Tehran city, under the coverage of Shahid Beheshti University of Medical Sciences. In this area, three medical health centers with field data on more than 90% of all covered families were chosen. An initial sample of 15,005 participants aged ≥ 3 years was selected by a multistage stratified cluster sampling method for the TLGS [16]. Among 10,368 subjects aged ≥ 20 years, those participants who had serum samples for measuring thyroid function were chosen to participate in the TTS.

For the present study, the inclusion criteria were as follows: (1) adults aged ≥ 20; and (2) individuals with normal thyroid function. On the other hand, subjects were excluded if they had a TSH < 0.32 mIU/L or a TSH > 5.06 mIU/L [17]. Those with levothyroxine, antithyroid drug, or corticosteroid usage, a history of thyroid surgery, thyroid radiation, or pregnant women were also excluded. Ninety-eight individuals lacked data necessary to categorize obesity phenotypes. Finally, 2988 subjects were included. Ultimately, 1360 males and 1628 females were included in our study. All individuals participated in the survey voluntarily and written informed consent was obtained from all subjects. The protocol of this study was approved by the ethics committee of Research Institute for Endocrine Sciences (RIES) of Shahid Beheshti University of Medical Sciences (Code: IR.SBMU.ENDOCRINE.REC.1400.117).

### Anthropometric measurements

The participants invited to the TTS were referred to trained physicians after signing an informed consent form. Participants wore light clothing and no shoes during anthropometric measurements. Weight and height were determined using a digital electronic weighing scale (Seca 707; range 0.1–150 kg; Seca, Hanover, MD) with an accuracy of up to 100 g and a tape meter stadiometer, respectively. In order to calculate body mass index (BMI), weight (kg) was divided by height (meters) squared. We measured the waist circumference (WC) in centimeters at the level of the umbilicus.

### Measurements of metabolic indices

Blood samples were taken between 7:00am and 9:00am from all study participants, following an overnight fast of 12–14 h. Utilizing glucose oxidase and enzymatic colorimetry, fasting glucose levels were measured. Serum total cholesterol (TC) and triglycerides (TGs) were determined using the enzymatic calorimetric method with cholesterol esterase and cholesterol oxidase and glycerol phosphate oxidase, respectively. High-density lipoprotein cholesterol (HDL-C) was measured after precipitation of the apolipoprotein B-containing lipoproteins with phosphotungistic acid. All these biochemical tests were conducted on the day of sampling, using commercial kits (Pars Azmoon, Inc., Tehran, Iran) by the Selectra 2 auto-analyzer (Vital Scientific, Spankeren, The Netherlands). Both inter- and intra-assay coefficients of variation (CVs) were < 2.3% for glucose, < 2.1% for TG, < 2% for TC, and < 3% for HDL-C.

Levels of FT4 and TSH were determined in − 70 °C stored serum samples by the electrochemiluminescence immunoassay method using Roche Diagnostics kits and a Roche/Hitachi Cobas e-411 analyzer (Mannheim, Germany). Lyophilized quality control material (Lyphochek Immunoassay plus Control; Bio-Rad Laboratories, Hercules, CA) was used to monitor the accuracy of the assay. The intra- and inter-assay CVs were 1.3% and 3.7% for FT4 and 1.5% and 4.5% for TSH determinations, respectively. Thyroid peroxidase antibodies (TPOAb) were assayed by an immunoenzymometric assay kit (IEMA; Monobind, Costa Mesa, CA) and the Sunrise ELISA reader (Tecan Co., Salzburg, Austria); intra- and inter-assay CVs were 3.9% and 4.7%, respectively. All measurements were carried out simultaneously in RIES's research laboratory.

The systolic blood pressure (SBP) and diastolic blood pressure (DBP) of subjects were measured twice in a seated position after 15 min of rest. First, a mercury sphygmomanometer was used to determine peak inflation levels. The average of two measurements was used to calculate the participant's blood pressure in this study.

### Definition of variables

The reference ranges were 0.32–5.06 mIU/L for TSH, and 0.91–1.55 pmol/L for FT4. Euthyroidism is defined as serum TSH and FT4 levels within the reference ranges.

Overweight/obesity was described as BMI ≥ 25 kg/m^2^ [18]. Joint Interim Statement (JIS) criteria were used to define abnormal metabolic components [17], (i) serum TG ≥ 150 mg/dL or taking lipid-lowering drugs; (ii) HDL-C < 40 mg/dL in men and < 50 mg/dL in women, or taking lipid-lowering drugs; (iii) systolic blood pressure (SBP) ≥ 130 mmHg or diastolic blood pressure (DBP) ≥ 85 mmHg, or taking antihypertensive drugs; and (iv) fasting blood glucose ≥ 100 mg/dL or undergoing treatment for diabetes. Individuals with < 2 JIS components were considered metabolically healthy, while the metabolically unhealthy group includes those who meet two or more criteria. Due to the high correlation between WC and BMI, it was excluded from the definition of metabolically unhealthy individuals [19].

Based on the BMI and metabolic state, participants were divided into four groups: (1) metabolically healthy normal weight (MHNW) defined as BMI < 25 kg/m^2^ and healthy metabolic status; (2) metabolically healthy overweight/obese (MHO) defined as BMI ≥ 25 kg/m^2^ and healthy metabolic status; (3) metabolically unhealthy normal weight (MUNW) defined as BMI < 25 kg/m^2^ and unhealthy metabolic status; (4) metabolically unhealthy overweight/obese (MUO) defined as BMI ≥ 25 kg/m^2^ and unhealthy metabolic status.

### Statistical analysis

All statistical analyses were performed using Stata version 15.1 statistical software (StataCorp LLC, Texas, USA). Data with a normal distribution were presented as the mean ± standard deviation, and data with a skewed distribution were presented as the median (25th percentile, 75th percentile). Categorical variables were represented by numbers (percentages). The difference in continuous variables was compared through one-way analysis of variance or Kruskal–Wallis one-way analysis of variance. Comparison between groups were conducted using chi-square tests or Fisher's exact tests for categorical variables. The statistical differences between thyroid hormones between different obesity phenotypes according to age and sex was compared using analysis of covariance (ANCOVA). Bonferroni was used for pairwise comparisons of the values between the different phenotype groups; *P* < 0.05 was considered statistically significant.

## Results

Totally, 2988 subjects with a mean (SD) age of 37.58 (12.21) were included in the present study. General characteristics of participants according to the obesity phenotypes are presented in Table 1. There was a significant difference between subgroups of obesity phenotype in gender, age, smoking status, BMI, WC, TC, TG, HDL, LDL, SBP, DBP, FPG, Cr, and eGFR (*P* < 0.01). The level of physical activity was not different among various obesity phenotypes (*P* = 0.273).

**Table 1:** General characteristics of study population according to different metabolic phenotypes

Variable	Total (*n* = 2988)	MHNW (*n* = 868)	MUNW (*n* = 363)	MHO (*n* = 660)	MUO (*n* = 1097)	*P* value
Gender, *n* (%)						
Male	1360 (45.5)	391 (45.0)	231 (63.6)	211 (32.0)	527 (48.0)	< 0.001
Female	1628 (54.5)	477 (55.0)	132 (36.4)	449 (68.0)	570 (52.0)	
Age, years	37.58 (12.21)	32.05 (11.40)^bcd^	39.43 (12.82)^acd^	37.12 (10.60)^abd^	41.63 (11.80)^abc^	< 0.001
BMI, kg/m^2^	26.28 (4.49)	21.83 (2.11)^bcd^	22.99 (1.73)^acd^	28.66 (3.27)^abd^	29.46 (3.36)^abc^	< 0.001
WC, cm	86.41 (11.77)	75.46 (7.22)^bcd^	80.82 (6.86)^acd^	90.06 (9.47)^abd^	94.73 (9.19)^abc^	< 0.001
TC, mg/dL	199.21 (41.55)	178.58 (34.46)^bcd^	200.92 (39.84)^ad^	198.50 (35.91)^ad^	215.40 (43.18)^abc^	< 0.001
TG, mg/dL	135.0 (90.0, 194.0)	86.0 (65.0, 110.7)^bcd^	179.5 (154.0, 231.0)^acd^	106.0 (82.0, 132.0)^abd^	195.0 (158.0, 250.5)^abc^	< 0.001
HDL, mg/dL	41.93 (11.07)	47.55 (10.52)^bd^	35.73 (7.51)^ac^	46.46 (11.45)^bd^	36.81 (8.52)^ac^	< 0.001
LDL, mg/dL	126.56 (35.29)	112.60 (31.33)^bcd^	126.01 (33.75)^ac^	129.65 (31.32)^ab^	136.47 (37.49)^abc^	< 0.001
SBP, mmHg	114.78 (15.52)	107.58 (10.93)^bcd^	115.68 (15.53)^acd^	111.12 (12.14)^abd^	122.43 (16.91)^abc^	< 0.001
DBP, mmHg	76.01 (10.25)	70.77 (8.07)^bcd^	76.11 (10.28)^acd^	74.26 (8.46)^abd^	81.21 (10.30)^bc^	< 0.001
FPG, mg/dL	88.99 (8.95)	85.72 (6.96)^bcd^	89.71 (9.59)^acd^	87.14 (7.26)^abd^	92.45 (9.79)^abc^	< 0.001
Cr, μmol/L	1.03 (0.15)	1.02 (0.15)^bd^	1.06 (0.14)^ac^	1.02 (0.14)^bd^	1.05 (0.15)^ac^	< 0.001
eGFR, mL/min/1.73	80.05 (12.28)	84.57 (12.31)	80.37 (12.71)	78.34 (10.87)	77.39 (11.90)	< 0.001
Smoking, *n* (%)						
Current	383 (12.8)	115 (13.3)	61 (16.8)	60 (9.1)	147 (13.4)	0.003
Former or never	2598 (87.2)	749 (86.7)	302 (83.2)	599 (90.9)	948 (86.6)	
Physical activity, *n* (%)						
Low	2032 (68.4)	589 (68.2)	233 (64.4)	448 (68.3)	762 (69.9)	0.273
High	940 (31.6)	275 (31.8)	129 (35.6)	208 (31.7)	328 (30.1)	

Continuous variables with normal distribution were reported as mean ± SD; Continuous variables with non-normal distribution were reported as median (interquartile range); Categorical variables were reported as *n* (%)

MHNW, metabolically healthy normal weight; MHO, metabolically healthy overweight/obese; MUNW, metabolically unhealthy normal weight; MUO, metabolically unhealthy overweight/obese; BMI, body mass index; WC, waist circumference; TG, triglycerides; TC, total cholesterol; LDL, low density lipoprotein-cholesterol; HDL, high density lipoprotein-cholesterol; FPG, fasting plasma glucose; SBP, systolic blood pressure; DBP, diastolic blood pressure; FT4, free thyroxine; TPO-Ab, thyroid peroxidase antibody; Cr, creatinine; eGFR, estimated glomerular filtration rate

*Difference between groups, *P* value is reported based on the one-way ANOVA for normally distributed continuous variables, Kruskal–Wallis for non-normally distributed continuous variables, and Chi-square for categorical variables. A Bonferroni post hoc test was used to show differences between groups

^a^Significant difference with MHNW

^b^Significant difference with MUNW

^c^Significant difference with MHO

^d^Significant difference with MUO

In Table 2, the general characteristics of the study population are provided according to different age and sex groups. It was observed that younger men have a lower WC (*P* < 0.001), TC (*P* = 0.001), LDL (*P* < 0.001), SBP (*P* < 0.001), DBP (*P* = 0.002), FPG (*P* < 0.001), and TPO Ab (*P* < 0.001). In contrast, higher FT4, Cr, GFR, and physical activity were obvious in under 55 years men whe compared with ≥ 55 years (*P* < 0.05). In younger women, the mean of BMI, WC, TC, TG, HDL, LDL, SBP, DBP, FPG, and physical activity were lower when compared with ≥ 55 years (*P* < 0.05). However, older women had a lower level of TSH, Cr, and eGFR (*P* < 0.05). Smoking status was not different in none of genders according to age (*P* > 0.05).

**Table 2:** General characteristics of study population according to different age and sex groups

Variable	Men < 55 years (*n* = 1178)	Men ≥ 55 years (*n* = 182)	*P* value	women < 55 years (*n* = 1498)	women ≥ 55 years (*n* = 130)	*P* value
BMI, kg/m^2^	25.64 (4.16)	25.93 (4.16)	0.377	26.61 (4.68)	28.91 (4.37)	< 0.001
WC, cm	87.85 (11.30)	91.24 (10.44)	< 0.001	84.07 (11.75)	93.67 (10.99)	< 0.001
TC, mg/dL	196.46 (39.97)	207.34 (39.77)	0.001	196.68 (39.60)	241.93 (53.83)	< 0.001
TG, mg/dL	158.0 (103.0, 217.0)	148.5 (97.0, 213.25)	0.323	115.0 (80.0, 172.0)	160.0 (121.0, 208.5)	< 0.001
HDL, mg/dL	37.82 (9.31)	38.77 (9.36)	0.202	45.10 (11.39)	47.11 (10.31)	0.037
LDL, mg/dL	124.63 (34.66)	134.60 (33.01)	< 0.001	124.50 (34.24)	157.48 (40.53)	< 0.001
SBP, mmHg	114.94 (13.08)	128.80 (20.20)	< 0.001	111.31 (13.75)	134.01 (21.45)	< 0.001
DBP, mmHg	76.00 (10.27)	79.01 (12.36)	0.002	75.08 (9.66)	82.68 (10.25)	< 0.001
FPG, mg/dL	89.73 (8.74)	92.47 (9.75)	< 0.001	87.67 (8.74)	92.64 (9.25)	< 0.001
TSH, mIU/L	1.43 (0.94, 2.06)	1.33 (0.83, 2.11)	0.144	1.77 (1.13, 2.67)	1.34 (0.91, 2.12)	< 0.001
FT4, ng/dL	1.26 (0.14)	1.20 (0.14)	< 0.001	1.18 (0.14)	1.19 (0.14)	0.474
TPO-Ab, IU/mL	4.73 (2.88, 8.99)	6.12 (3.97, 10.03)	< 0.001	5.63 (3.25, 11.48)	6.44 (3.72, 12.55)	0.163
Cr, μmol/L	1.15 (0.12)	1.13 (0.10)	0.001	.94 (0.09)	.93 (0.07)	0.017
eGFR, mL/min/1.73	83.11 (12.38)	69.74 (7.87)	< 0.001	80.01 (11.63)	67.24 (6.16)	< 0.001
Smoking, *n* (%)						
Current	304 (25.9)	37 (20.3)	0.127	38 (2.5)	4 (3.1)	0.935
Former or never	869 (74.1)	145 (79.7)		1458 (97.5)	126 (69.6)	
Physical activity, *n* (%)						
Low	813 (69.5)	112 (61.9)	0.048	1007 (67.4)	100 (77.5)	0.024
High	356 (30.5)	69 (38.1)		486 (32.6)	29 (22.5)	

Continuous variables with normal distribution were reported as mean ± SD; continuous variables with non-normal distribution were reported as median (interquartile range); Categorical variables were reported as *n* (%)

MHNW, metabolically healthy normal weight; MHO, metabolically healthy overweight/obese; MUNW, metabolically unhealthy normal weight; MUO, metabolically unhealthy overweight/obese; BMI, body mass index; WC, waist circumference; TG, triglycerides; TC, total cholesterol; LDL, low density lipoprotein-cholesterol; HDL, high density lipoprotein-cholesterol; FPG, fasting plasma glucose; SBP, systolic blood pressure; DBP, diastolic blood pressure; FT4, free thyroxine; TPO-Ab, thyroid peroxidase antibody; Cr, creatinine; eGFR, estimated glomerular filtration rate

*Difference between groups, *P* value is reported based on the independent sample *t* test for continuous variables and Chi-square for categorical variables

The parameters of thyroid function are compared according to different obesity phenotypes in Table 3. It was found that metabolically healthy normal weight participants had higher levels of FT4 when compared with metabolically healthy or unhealthy obese subjects (*P* < 0.001). These differences remained significant after adjustment for the effect of age, waist circumference, smoking, and physical activity. No difference was observed in the levels of TSH (*P* = 0.260) and TPO-Ab (*P* = 0.944) among obesity phenotypes. In the subgroup analysis according to the age, adjusted for the effect of age, waist circumference, smoking, and physical activity, a significant difference was observed in the level of FT4 only in subjects with age < 55 years (*P* = 0.001) (Table 4). However, analyzing men and women separately did not show a significant difference in the FT4 level among obesity phenotypes (*P* > 0.05) (Table 5). In addition, no difference was observed in the serum TSH level in subgroup analysis of age and gender in the adjusted model (*P* > 0.05).

**Table 3:** Comparison of thyroid hormone parameters in different obesity phenotypes

Variable	Total (*n* = 2988)	MHNW (*n* = 868)	MUNW (*n* = 363)	MHO (*n* = 660)	MUO (*n* = 1097)	*P* value^1^	*P* value^2^
TSH, mIU/L	1.55 (1.02, 2.36)	1.55 (1.01, 2.36)	1.51 (0.97, 2.44)	1.62 (1.1, 2.41)	1.52 (0.99, 2.33)	0.260	0.278
FT4, ng/dL	1.21 (0.14)	1.24 (0.14)^cd^	1.22 (0.14)^cd^	1.19 (0.14)^ab^	1.20 (0.14)^ab^	< 0.001	0.031
TPO-Ab, IU/mL	5.28 (3.14, 10.33)	5.42 (3.11, 10.58)	5.20 (3.17, 9.48)	5.13 (3.09, 10.43)	5.28 (3.15, 10.44)	0.944	0.4

^1^*P* value is reported based on the one-way ANOVA for normally distributed continuous variables and Kruskal–Wallis for non-normally distributed continuous variables. A Bonferroni post hoc test was used to show differences between groups

^a^Significant difference with MHNW

^b^Significant difference with MUNW

^c^Significant difference with MHO

^d^Significant difference with MUO

^2^ANCOVA adjusted for age, sex, waist circumference, smoking, and physical activity

**Table 4:** Comparison of thyroid hormone parameters in different obesity phenotypes according to age

Variable	MHNW (*n* = 868)	MUNW (*n* = 363)	MHO (*n* = 660)	MUO (*n* = 1097)	*P* value^1^
Age < 55 years					
TSH, mIU/L	1.57 (1.02, 2.36)	1.57 (1.03, 2.51)	1.64 (1.11, 2.43)	1.56 (1.00, 2.38)	0.312
FT4, ng/dL	1.25 (0.14)	1.23 (0.14)	1.19 (0.14)	1.20 (0.14)	0.001
Age ≥ 55 years					
TSH, mIU/L	1.45 (0.82, 2.27)	1.27 (0.73, 2.14)	1.26 (0.84, 2.03)	1.38 (0.90, 2.12)	0.631
FT4, ng/dL	1.19 (0.11)	1.20 (0.15)	1.21 (0.14)	1.19 (0.14)	0.689

^1^ANCOVA adjusted for age, waist circumference, smoking, and physical activity

**Table 5:** Comparison of thyroid hormone parameters in different obesity phenotypes according to sex

Variable	MHNW (*n* = 868)	MUNW (*n* = 363)	MHO (*n* = 660)	MUO (*n* = 1097)	*P* value^1^
Men					
TSH, mIU/L	1.42 (0.90, 1.98)	1.44 (0.95, 2.19)	1.40 (0.89, 1.95)	1.43 (0.94, 2.09)	0.414
FT4, ng/dL	1.28 (0.14)	1.25 (0.14)	1.24 (0.13)	1.24 (0.14)	0.120
Women					
TSH, mIU/L	1.73 (1.11, 2.70)	1.76 (1.03, 2.70)	1.78 (1.16, 2.66)	1.68 (1.06, 2.57)	0.378
FT4, ng/dL	1.21 (0.14)	1.19 (0.13)	1.17 (0.13)	1.16 (0.13)	0.167

^1^ANCOVA adjusted for age, waist circumference, smoking, and physical activity

## Discussion

Studying thyroid function and different obesity phenotypes based on the age and sex in the TTS was the purpose of this study. The MHNW participants had higher levels of FT4 than MHO or MUO participants, even after adjusting for age, WC, smoking, and physical activity. TSH levels did not differ between the groups.

A limited number of studies have examined this relationship with respect to different obesity phenotypes with inconsistent results [12–14]. In another study [20], fT4 levels were significantly associated with reduced MetS prevalence in the general population, in men, and in young subjects. Additionally, a significant negative relation was found between high WC and high TG with fT4 according to gender. Results from the study reported that serum TSH within the reference range was not related to MetS or any of its components in any group. Similarly, a low normal FT4 value was associated with an increased risk of MetS, according to Mehran et al. [21].

The thyroid hormone is responsible for regulating the basal energy expenditure and influencing glucose and lipid metabolism [22]. In light of this, many studies have shown an association between thyroid function with obesity and obesity-related states including hypertension, hyperglycemia, hyperlipidemia, and MetS [23–25]. A number of plausible mechanisms have been proposed. Low thyroid function may cause obesity by lowering the basal metabolic rate, similar to hypothyroidism's mechanisms [22]. Further evidence supports this finding, which also found that weight gain was correlated with slightly decreased thyroid function during a 5-year follow-up in 4082 participants [26]. There is also the possibility that obesity could alter thyroid hormone levels. There is evidence that obesity affects thyroid function by reducing T3 and TSH receptor expression in fat of obese individuals, which has been linked to thyroid insufficiency in the tissue [27]. The frequent observation that TSH levels are elevated during obesity is not regarded as a functional defect, but as a mere manifestation of a deranged hypothalamic–pituitary axis or an increase in leptin-induced TRH production and type 2 iodothyronine deiodinase (D2) inhibition in the thyrotrophs [28]. There are, however, conflicting findings regarding the relationship between TSH and FT4 with MetS and its components, mostly from cross-sectional studies. MetS has been reported to be a condition of sympathetic hyperactivity by Huggett et al. [29]. Sympathetic activation is elevated in obese humans, especially in those with abdominal obesity, due to insulin's stimulatory influence [30], which may be a factor in MetS development in obese people. As an adaptive thermogenic mechanism, Depegrola et al. found that progressive central fat accumulation was accompanied by an elevation in free triiodothyronine (T3) independent of insulin resistance [31]. Cross-sectional studies by Roos et al. [32], Lai et al. [33] and Mehran et al. [34] did not find an association between TSH and MetS in euthyroid individuals. Disparities between the findings can be explained by changes in inclusion criteria, settings, ethnicity, adjustments made to analyses, and the population's iodine status.

In spite of TSH's reputation as a highly sensitive thyroid disorder marker, FT4 may offer a more accurate indication of thyroid function, since FT4 uptake in the blood, its deiodination into active T3, and T3 binding to nuclear receptors are all determined by circulating FT4 [35].

It is known that obesity itself can change thyroid hormone levels, so the associations observed in this cross-sectional study may have primarily been due to obesity. As a result of a decrease in thyroid hormone receptors in obese people, both TSH and peripheral thyroid hormone levels increase [36], which leads to reduction in negative feedback between them. As leptin levels increase in adipose tissue, the hypothalamus–pituitary–thyroid axis gets stimulated, causing thyrotropin levels to increase. Following weight loss, obese subjects undergo an adaptation process that increases their energy expenditure as TSH levels normalize [37].

The study's strengths are the large healthy euthyroid population, the confounding variables have been minimized, suitable exclusion criteria have been employed, and FT4 and TSH testing were performed. However, because of cross-sectional nature of this study, causality cannot be inferred. A number of geographic and demographic factors, as well as different definitions of euthyroidism, lead to the inability to generalize these results to other populations. Moreover, urinary iodine levels were not measured in our study; thus, iodine intake could have biased our results. The third limitation in this study is that the nutritional status was not assessed in detail, which may lead to potential biases.

## Conclusion

The results reveal that low normal FT4 levels are independently associated with "metabolically unhealthy" phenotype in overweight/obese euthyroid individuals. By analyzing serum levels of thyroid hormones, we were able to objectively investigate the association between obesity or a metabolic phenotype in individuals with normal thyroid function. There is a need for further research into the mechanisms by which low FT4 levels can contribute to a metabolically unhealthy state despite being in the euthyroid range.

## Data Availability

The data that support the findings of this study are available on request from the corresponding author, MV. The data are not publicly available due to restrictions e.g. their containing information that could compromise the privacy of research participants.
